# Hepatitis B Vaccines and HPV Vaccines Have Been Hailed as Major Public Health Achievements in Preventing Cancer—Could a Schistosomiasis Vaccine be the Third?

**DOI:** 10.1371/journal.pntd.0003598

**Published:** 2015-05-28

**Authors:** Michael H. Hsieh, Julia M. L. Brotherton, Afzal A. Siddiqui

**Affiliations:** 1 Biomedical Research Institute, Rockville, Maryland, United States of America; 2 Children’s National Health System, Washington, DC, United States of America; 3 The George Washington University, Washington, DC, United States of America; 4 National HPV Vaccination Program Register, East Melbourne, Victoria, Australia; 5 School of Global and Population Health, University of Melbourne, Melbourne, Australia; 6 Center for Tropical Medicine and Infectious Diseases, Texas Tech University Health Sciences Center, Lubbock, Texas, United States of America; Lindsley F. Kimball Research Institute, UNITED STATES

Urogenital schistosomiasis affects 112 million people, mostly in sub-Saharan Africa, the Middle East, and, most recently, Corsica [1,2]. This disease is spreading to new areas despite efforts to eradicate the intermediate snail host, improved sanitation infrastructure, and implementation of mass drug administration using praziquantel. No approved human vaccine exists to prevent or treat schistosomiasis. People become infected with *Schistosoma haematobium*, the etiologic agent of urogenital schistosomiasis, when they are exposed to fresh water infested by *Bulinus* snails that release cercariae. After burrowing through the skin of human hosts, cercariae develop into egg-laying adults. The worms preferentially localize to the pelvic veins, where they lay eggs in the pelvic organs. Roughly half of the eggs are expulsed in urine and complete the life cycle when voided into fresh water. Eggs retained in the body are inflammatory, and are thought to trigger bladder cancer. This has parallels to certain human papillomavirus (HPV) types that also induce cancer through chronic infection of the genital tract. In addition, *S*. *haematobium* infection is also a common cause of genital morbidity in girls and adult women and it can impair female reproductive capacity.

We contend that a successful vaccine for urogenital schistosomiasis (whether specific only to *S*. *haematobium* or protective against multiple schistosome species, i.e., *S*. *mansoni* and *S*. *japonicum*) may confer major public health benefits by preventing bladder cancer. Given that both *S*. *haematobium* and HPV cause urogenital cancers, and an efficacious HPV vaccine has been successfully developed and implemented, we may be able to glean important lessons relevant to cancer prevention using a vaccine for urogenital schistosomiasis. Whilst the modes of transmission differ, both are infections with complex natural histories (that communities often have little understanding of) that can ultimately cause cancer. Both infections cause greatest disease burden in countries with limited health resources.

HPV vaccine delivery has been complicated by the need to deliver the vaccine to pre-adolescents, due to the vaccine being prophylactic and not having been trialed pre-licensure in children. Delivery has, thus, been challenging due to the lack of routine vaccination programs for this age group. It has been most successfully delivered by using a school-based approach to vaccination and a campaign-style program, similar to that used in many countries for other vaccines, to provide vaccines to those children outside of school [3]. For urogenital schistosomiasis, the infrastructure for mass drug administration campaigns, many of which are school-based, provides a parallel opportunity to access the population to be vaccinated and to offer vaccination along with treatment. Importantly, data from nonhuman primate trials of the leading candidate vaccine suggest that it may feature both pre- and post-exposure efficacy for intestinal and urogenital schistosomiasis [4]. As the average age of first *S*. *haematobium* infection is early childhood, establishing the safety and efficacy of the vaccine in young children should be a priority. The requirement to receive three doses of HPV vaccine has complicated the achievement of high coverage. Thus, post-licensure research is focused on investigating one- or two-dose HPV vaccine schedules [5]. We believe that identification of optimal vaccine formulation and delivery methods/schedules for a schistosomiasis vaccine should be prioritized, noting that the HPV vaccine experience provides important information for understanding how to optimize immune response to subunit vaccines [3].

The HPV vaccination experience suggests that portraying an anti-infection vaccine as an anti-cancer vaccine produces high public acceptance of vaccination without the requirement for recipients to have a complex understanding of infection or transmission itself [6]. In HPV vaccine implementation, community mobilization and awareness raising about the vaccine, including proactive strategies and systems for addressing vaccine safety concerns, have emerged as important strategies prior to vaccination [7]. We anticipate that such strategies would also be useful for a urogenital schistosomiasis vaccine.

The greatest barrier for implementation of HPV vaccines in the developing world, where they could save the most lives, remains cost. New technology has facilitated the development of highly efficacious vaccines but we also need to harness scientific potential to produce affordable vaccines, preferably through nonprofit product development partnerships.

Some of the caveats to consider may include that direct parallels between a HPV and a *S*. *haematobium* vaccine may be difficult to draw across the board because HPV is a sexually transmitted disease, whereas *S*. *haematobium* is not. Furthermore, the HPV vaccine is primarily a prophylactic vaccine with no therapeutic effects, whereas the Sm-p80 vaccine is being developed as both a prophylactic and therapeutic vaccine [4].

In summary, we can usefully draw upon lessons learned from HPV vaccine, just as the HPV vaccine drew from the experience of the first anti-cancer vaccine, the hepatitis B virus vaccine, which prevents liver cancer [8]. In turn, vaccines against flukes other than schistosomes, such as *Opisthorchis* and *Clonorchis*, may also draw inspiration from the HPV vaccine, given the propensity of liver flukes to cause cholangiocarcinomas [9]. We believe that by learning from these lessons that a schistosomiasis vaccine could be the third successful anti-cancer vaccine with a significant public health impact.

## References

[1] Berry A, Mone H, Iriart X, Mouahid G, Abbo O, Boissier J, et al (2014) Schistosomiasis Haematobium, Corsica, France. Emerg Infect Dis J—CDC. 10.3201/eid2009.140928 PMC4178424 25153697

[2] Rinaldi G, Young ND, Honeycutt JD, Brindley PJ, Gasser RB, Hsieh MH (2014) New Research Tools for Urogenital Schistosomiasis. J Infect Dis: 1–29. 10.1093/infdis/jiu392 25240172 PMC4416124

[3] Karmakar S, Zhang W, Ahmad G, Torben W, Alam MU, Le L, et al (2014) Use of an Sm-p80-based therapeutic vaccine to kill established adult schistosome parasites in chronically infected baboons. J Infect Dis 209: 1929–1940. 10.1093/infdis/jiu031 24436452 PMC4038147

[4] Dobson SRM, McNeil S, Dionne M, Dawar M, Ogilvie G, Krajden M, et al (2013) Immunogenicity of 2 doses of HPV vaccine in younger adolescents vs 3 doses in young women: A randomized clinical trial. Jama 309: 1793–1802. 10.1001/jama.2013.1625 23632723

[5] Schiller JT, Lowy DR (2014) Raising Expectations For Subunit Vaccine. J Infect Dis. 10.1093/infdis/jiu648 PMC4400525 25420478

[6] LaMontagne DS, Barge S, Le NT, Mugisha E, Penny M, Gandhi S, et al (2011) Human papillomavirus vaccine delivery strategies that achieved high coverage in low- and middle-income countries. Bull World Health Organ 89: 821–830. 10.2471/BLT.11.089862 22084528 PMC3209730

[7] Bingham A, Drake JK, LaMontagne DS (2009) Sociocultural issues in the introduction of human papillomavirus vaccine in low-resource settings. Arch Pediatr Adolesc Med 163: 455–461. 10.1001/archpediatrics.2009.50 19414692

[8] Kane MA (2010) Global implementation of human papillomavirus (HPV) vaccine: Lessons from hepatitis B vaccine. Gynecol Oncol 117. 10.1016/j.ygyno.2010.01.029 20129654

[9] Sripa B, Brindley PJ, Mulvenna J, Laha T, Smout MJ, Mairiang E, et al (2012) The tumorigenic liver fluke Opisthorchis viverrini—multiple pathways to cancer. Trends Parasitol 28: 395–407. 10.1016/j.pt.2012.07.006 22947297 PMC3682777

